# Circadian rhythm disruption and mental health

**DOI:** 10.1038/s41398-020-0694-0

**Published:** 2020-01-23

**Authors:** William H. Walker, James C. Walton, A. Courtney DeVries, Randy J. Nelson

**Affiliations:** 1 Department of Neuroscience, Rockefeller Neuroscience Institute West Virginia University, Morgantown, WV 26506 USA; 2 Department of Medicine, West Virginia University, Morgantown, WV 26506 USA

**Keywords:** Neuroscience, Psychiatric disorders

## Abstract

Circadian rhythms are internal manifestations of the solar day that permit adaptations to predictable environmental temporal changes. These ~24-h rhythms are controlled by molecular clockworks within the brain that are reset daily to precisely 24 h by exposure to the light–dark cycle. Information from the master clock in the mammalian hypothalamus conveys temporal information to the entire body via humoral and neural communication. A bidirectional relationship exists between mood disorders and circadian rhythms. Mood disorders are often associated with disrupted circadian clock-controlled responses, such as sleep and cortisol secretion, whereas disruption of circadian rhythms via jet lag, night-shift work, or exposure to artificial light at night, can precipitate or exacerbate affective symptoms in susceptible individuals. Evidence suggests strong associations between circadian rhythms and mental health, but only recently have studies begun to discover the direct interactions between the circadian system and mood regulation. This review provides an overview of disrupted circadian rhythms and the relationship to behavioral health and psychiatry. The focus of this review is delineating the role of disruption of circadian rhythms on mood disorders using human night shift studies, as well as jet lag studies to identify links. We also review animal models of disrupted circadian rhythms on affective responses. Lastly, we propose low-cost behavioral and lifestyle changes to improve circadian rhythms and presumably behavioral health.

## Introduction

Life on this planet is adapted to the 24-hour (h) solar day. Over evolutionary time, the predictable daily cycles of light and dark have been internalized in the form of circadian rhythms. Circadian (*circa* = about; *dies* = day) rhythms allow synchronization of biological and behavioral processes to the external temporal environment. Thus, optimal timing of physiological events is coordinated by these internal timekeepers. Endogenous circadian rhythms have a period of ~24 h and are reset on a daily basis to precisely 24 h through exposure to light–dark cues. Over the past century, however, the boundaries between day and night have been obscured by the widespread adoption of electric light at night. As a result, behavioral health and psychiatric consequences of circadian disruption by light at night are becoming increasingly apparent^1,2^. The focus of this review is delineating the role of disruption of circadian rhythms on mood disorders using human night shift studies, as well as jet lag studies to identify links. We also review animal models of disrupted circadian rhythms on affective responses. Finally, we propose low-cost behavioral and lifestyle changes to improve circadian rhythms and presumably behavioral health.

Before electric lights were introduced about a century ago, people were exposed to minimal light at night. A full moon on a clear night illuminates the temporal zone environment 0.1–0.3 lux^3^, or up to 1.0 lux in the tropics^4^. From a meter in distance, a single candle only produces ~1.0 lux of light. The invention of electric lights near the end of the 19th century was a welcomed advance and their popularity grew rapidly^5^. For the first time in history, humans could extend the day resolving their fears of night-time crime, fire, and the supernatural^5^.

The economic benefits of electric lighting soon ensued as night shift work was introduced. By the end of the 20th century, technology provided people with additional sources of night-time light, including television, computer screens, e-readers, smartphones, and tablet computers. Today, >80% of humans and 99% of those living in the US or Europe experience significant night-time light pollution^6^; indeed, the artificial night sky glow is sufficiently bright that two-thirds of Europeans and nearly 80% of North Americans cannot see the Milky Way^6^. Importantly, between 15 and 20% of people in industrial societies work night shifts; these individuals serve as the proverbial ‘canaries in a coal mine’ for how disrupted circadian rhythms affect health. Night shift workers suffer several health disparities compared to their day shift worker counterparts; e.g., night shift workers display increased rates of several types of cancer, elevated incidence of cardiovascular and metabolic disorders, as well as increased prevalence of behavioral health and psychiatric disorders^7–9^. Even those who do not work night shifts are exposed to nightly light pollution from other sources^9,10^. Light intensities from an average urban street ranges between 5–15 lux and a typical living room is illuminated nightly between 100–300 lux; electronic tablet computers emit ~40 lux, depending on the size of the screen^10^. Remarkably, 36% of adults and 34% of children sleep with a light-producing electronic device, such as a television or computer^11^. Again, pervasiveness and intensity of nighttime light exposure is unprecedented in our history.

Exposure to light at night perturbs the circadian system because light is the major entraining cue used by the body to discriminate day from night. When exposure to light is mistimed or nearly constant, biological and behavioral rhythms can become desynchronized, leading to negative consequences for health. The relationship among mood disorders, light, and circadian rhythms have long been recognized^9^. One example is seasonal affective disorder in which mood oscillates between dysthymia during the short day lengths of winter and euthymia during the long summer days. Indeed, many mood disorders are either characterized by sleep and circadian rhythm disruption or precipitated by an irregular light–dark cycle. Sleep disruption is a diagnostic criterion for major depression, bipolar disorder, post-traumatic stress disorder, generalized anxiety, and other mood disorders^12^.

Shift work disorder (SWD) is a circadian rhythm sleep disorder associated with working outside of the typical 800 to 1700 h shifts^13^. Individuals with SWD report insomnia, difficulty falling asleep, and experiencing excessive sleepiness or micro-naps when it is important to be alert and productive^14^. Affective responses associated with SWD include irritability, depression, and difficulty maintaining personal relationships. Shift work disorder can be provoked by night shifts, rotating shifts, afternoon shifts, or even early morning shifts. SWD is associated with chronic sleep deprivation and a persistent “sleep debt”^15^. Such chronic sleep loss has serious consequences for health, productivity, and safety. Pharmacological treatments for SWD have largely been ineffective^16^, although proper timing of caffeine has been reported to improve alertness for people working simulated night shifts^17^. Melatonin appears to improve adaptation to day-time sleep schedules and increases the length of sleep^16,17^. However, separating out the effects of circadian rhythm disruption from sleep disruption on mood disorders often requires studies on nocturnal animals as most studies on humans cannot parse the two factors^9^.

In addition to light at night, circadian rhythms can be disrupted by another modern convenience, jet travel across time zones. Jet lag, also called jet lag disorder, is a transient sleep problem that arises when an individual travels across multiple time zones^18^. Because circadian rhythms do not instantaneously reset, for several days they may remain more closely entrained to the original time zone than the current time zone; the lag in synchronizing these internal rhythms to the current photic (light) and non-photic (social interactions, timing of meals, etc.) cues results in disturbed sleep, daytime fatigue, hormone profiles, gastrointestinal issues, and changes in mood. These symptoms are all manifestations of a misaligned circadian system. People who regularly cross multiple time zones, such as international flight crews, often report persistent jet lag symptoms; negative symptoms typically increase with the number of time zones crossed and are worse for travelers flying in an eastern direction than a western direction due to the necessity to advance the body’s clock^19,20^. Several studies have suggested that mood changes, especially dysphoric mood, are an important aspect of jet lag^21^. For example, a study of five men experiencing 7-h phase shifts (one westward shift and one eastward shift) over the course of a month reported that the eastward shift significantly disrupted sleep and elevated anxiety and depression scores^20^.

Although scientific studies have focused primarily on the health consequences of “jet lag” caused by air travel across time zones, a much greater proportion of the population regularly experiences ‘social jet lag’, which is a related phenomenon in which individuals remain in their time zone but significantly shift their sleep-wake patterns several days per week^22^. Indeed, social influences on sleep time commence as children begin day care or school— parents often enforce a set bedtime and awakening during the school week, but then allow their children stay up later and sleep later to resolve sleep debts on weekends and vacations^22^. A large scale epidemiological study confirmed that both sleep timing and duration are substantially challenged by work and school schedules or other social events. To align sleep and wake times with social obligations, 80% of the population uses alarm clocks on school or work days^23^. Early school and work schedules are particularly difficult for individuals with late chronotypes (i.e. “evening larks” with late sleep periods).

Social influences on the timing of human sleep becomes apparent when comparing sleep–wake behavior on work and free days. The vast majority of people display clear differences in both sleep duration and timing; sleep duration shortens significantly (by ~2 h/night) from the age of 10–17^22^, reflecting reduced sleep during school or work days. The profound mismatch between the “enforced” sleep duration on school/work days and the variable sleep duration on days off demonstrates the strong effect of social times on daily sleep behavior. Although only ~20% of individuals in Europe and North America work night shifts, and a much smaller proportion of individuals experience chronic jet lag, virtually everyone experiences circadian disruption provoked by social jet lag, and the associated change in the timing of light exposure.

In sum, lifestyle changes that have occurred in response to technological advances over the past century have resulted in unprecedented changes in the timing and duration of light exposure, which in turn has the potential to desynchronize circadian rhythms; increased prevalence of disrupted circadian rhythms strongly correlates with the increased incidence of mood disorders^9,24–26^. Notably, neural systems associated with affect, such as the limbic brain regions, monoamine neurotransmitters, and the hypothalamic–pituitary–adrenal axis (HPA), are all under circadian regulation^27–31^. Thus, it is reasonable to expect that widespread disruption of circadian rhythms would contribute to the prevalence of mood disorders^9,32,33^.

### Light detection and circadian rhythms

In addition to the image forming photoreceptors (rods and cones) in the retina, light is also detected by specialized photoreceptor cells in mammals. This third class of photoreceptors, called intrinsically photosensitive retinal ganglion cells (ipRGCs), are engaged in several non-image-forming functions, including circadian phototransduction^34^. The ipRGCs constitute a small fraction of the larger class of retinal ganglion cells, but their expression of melanopsin, a photopigment, makes them exquisitely photosensitive^34,35^. Blue light (~480 nm) most strongly stimulates the melanopsin photopigment ipRGCs, whereas red light (>600 nm) has minimal effect^35^. The characteristics of sunlight change across the day and in response to the atmosphere; typically, sunlight contains more short (blue) wavelengths during the day and then shifts to longer (red) wavelengths toward sunset. The sensitivity spectrum of melanopsin may be an adaptation to the natural solar cycle, so that ipRGCs are tuned to discriminate daylight from evening, allowing better entrainment of circadian rhythms^9^. When exposed to light in the activating spectrum, ipRGCs propagate neural impulses via the retinohypothalmic tract directly to the circadian master clock, the mammalian suprachiasmatic nucleus (SCN) of the hypothalamus. This monosynaptic pathway conveys photic information via the release of excitatory amino acids, namely, glutamate. Activation of the SCN via light results in Ca^2+^ influx and activation of intracellular signaling cascade; Ultimately, leading to increased expression of Period genes to set the molecular clock^36^. In addition, ipRGCs have other direct and indirect targets throughout the brain, including mood-related structures (e.g. amygdala and habenula)^37^.

The SCN molecular clock comprises a set of transcriptional–translational feedback loops that drive rhythmic 24-h expression of the core canonical clock components^38,39^. In the primary feedback loop, circadian locomotor output cycle kaput (*CLOCK*) and brain and muscle ARNT-like protein 1 (*BMAL1*) proteins form heterodimers in the cytosol of cells of the SCN. The CLOCK-BMAL1 complex translocates to the nucleus and binds to regulatory elements of the DNA containing E-boxes, which in turn activate expression of period (*PER1, PER2, and PER3*) and cryptochrome *(CRY1 and CRY 2)* genes. PER and CRY proteins then heterodimerize and translocate into the nucleus, where they repress their own transcription by acting on the CLOCK-BMAL1 complexes. In mice, activation of CLOCK-BMAL1 occurs in early morning leading to the transcription of *PER* and *CRY* protein in the early afternoon and subsequent repression of CLOCK-BMAL1 transcription in the evening/night^39^. In an interacting feedback loop, CLOCK-BMAL1 complexes activate expression of nuclear receptors, *REV-ERB α* and *RORα*. Their protein products feedback to regulate *BMAL1* by competitively binding retinoic acid-related orphan receptor response elements in the *BMAL1* promoter^40^. *REV-ERB*s repress the transcription of *BMAL1*, whereas RORs activate it. These two loops form the basis of the molecular clock, but a complex network of interacting genes and post-translational modifications ensure that the process takes ~24 h to complete^38,39^. This transcriptional–translational feedback loop is the basis of the intrinsic daily circadian rhythm.

In the absence of environmental signals, the molecular clock will continue to produce ~24-h or circadian rhythms. People living in caves or bunkers without light or temperature cues will maintain circadian sleep–wake and body temperature rhythms, although they begin to free-run with a rhythm slightly longer than 24 h^41^. Therefore, light serves as the major entraining (synchronizing) cue to precisely maintain synchrony with the environment. Light exposure during the night phase shifts the clock transcription-translations cycle by rapidly inducing expression of *Per1* or *Per2*, depending on whether the light occurs during the early or late night^42–44^. Phase shifting can be useful for adapting to changing seasonal day lengths, or more recently travel to a new time zone, but abrupt changes or inappropriate timing of light exposure can be problematic for daily life. For example, the use of electronics during the night can unintentionally phase shift the circadian rhythm, leaving it decoupled from the natural environmental light–dark cycle^45^, with potentially dysregulated physiology and behavior.

The SCN functions as the central circadian oscillator, although similar intracellular clock mechanisms are expressed in other brain regions, as well as in peripheral tissues. Clocks throughout the body remain synchronized with one another by responding to signals from the SCN, either through direct neural inputs or indirect signals such as humoral, behavioral, or other physiological rhythms. For example, timing of feeding and body temperature can help synchronize peripheral oscillators^46–48^. Aberrant light exposure that disrupts these rhythms may drive downstream dysregulation of circadian rhythms in peripheral systems.

The two most prominent endocrine manifestations of circadian rhythms are the daily cycles of melatonin and glucocorticoids (cortisol in humans; corticosterone in most rodents). Time of day information is transmitted to the pineal gland from the SCN to regulate production and secretion of the indole amine, melatonin. Melatonin is derived from serotonin via two enzymatic steps, and then secreted from the pineal gland at night in both diurnal and nocturnal animals. Circulating melatonin is also a cue to help entrain clocks in peripheral organs via several interactions with the molecular clock mechanism, including phase-resetting clock genes^49^. Cells in the periphery exposed to melatonin respond in ‘night-mode’, whereas cells not exposed to melatonin respond in ‘day-mode’. The secretion of melatonin is tightly controlled by light; <3 lux of light exposure at night is effective in suppressing the onset of melatonin secretion and shortens melatonin secretion duration in humans^50,51^. Thus, via suppression of melatonin rhythm, light at night has the potential to substantially alter physiology and behavior.

Additionally, the circadian system regulates glucocorticoid secretion from the adrenal glands, such that concentrations tend to peak in the morning just before awakening and decrease throughout the day in diurnal animals, including humans^52,53^. The reverse pattern occurs in nocturnal mammals. The functional significance of increased glucocorticoid concentrations during the active period and decreased concentrations during sleep is related to the hormone’s important role in glucose homeostasis. Glucocorticoids also subserve several physiological and behavioral responses to stress. Given the wide range of glucocorticoid effects on biological processes that are crucial for survival, it should not be surprising that glucocorticoid concentrations are tightly regulated by negative feedback at each level of the HPA axis. Indeed, dysregulated glucocorticoid secretion is associated with several debilitating health conditions, including major depressive disorder^54^. Because light exposure is an important zeitgeber for the circadian rhythm of cortisol in humans, exposure to light at night could dysregulate the HPA axis, in turn increasing the prevalence of cortisol-associated mood disorders^55^.

### Circadian rhythm disruption

Most of the evidence supporting the effects of atypical light exposure on affective responses has been gleaned from animal models, particularly rodents, because of the ease with which light exposure can be controlled. One advantage of using rodents is that the most common lab species are nocturnal, and so exposure to light at night occurs during their active and awake phase. Thus, light at night does not directly alter sleep in nocturnal species. This is important because most effects of disrupted circadian rhythms on human mood are attributed to sleep disruption, but studies using nocturnal species demonstrate that this is not the only cause as sleep remains intact when animals are exposed to dim light at night^56^. Studies of light at night exposure in diurnal rodent species generally yield similar affective responses to nocturnal rodents^57,58^. Another important difference between humans and individuals of some rodent species is the production of pineal melatonin. Although both nocturnal and diurnal species produce melatonin during the dark phase, several common laboratory strains of mice have no detectable pineal melatonin rhythms. Nonetheless, studies using Swiss Webster mice (which have no detectable pineal melatonin) vs. Siberian hamsters (which have a robust pineal melatonin rhythm) have reported similar effects of light at night on affective responses^33,59–61^. This observation suggests that suppressed melatonin by nighttime light is not the only or even primary mechanism, but still may be an important contributor in humans and a potential point of intervention.

Accumulating evidence demonstrates both direct and indirect connections between artificial light at night and mood regulation. First, exposure to light at night can directly affect mood through ipRGC projections to brain regions involved in affect^62,63^. Mice exposed to alternating cycles of 3.5 h light and 3.5 h dark (termed T7), which do not significantly affect cycling clock gene expression or sleep, develop depressive-like symptoms^63^. In contrast, mice lacking the melanopsin gene are protected against such depressive responses, suggesting the behavioral changes occur because ipRGCs project either directly or via the SCN to brain regions involved in mood regulation. In addition, there are several indirect pathways affected by light at night that may contribute to mood disturbances^37^.

### Circadian rhythm disruption and major depressive disorder

Major depressive disorder (MDD) is characterized by alterations in mood, typically increased sadness and/or irritability that is accompanied by at least one of the following psychophysiological symptoms: alterations in sleep, sexual desire, or appetite, inability to experience pleasure, slowing of speech or actions, crying, and suicidal thoughts^64^. Diagnosis of MDD requires these symptoms to persist for at least two weeks and interfere with normal daily functions^64^. MDD affects vast numbers of people worldwide; the Global Burden of Disease Consortium examined the global incidence and prevalence for 328 diseases in 195 countries from 1990–2016, MDD ranked within the top ten leading causes of disease burden in all but four countries examined^65^. The incidence of MDD worldwide is on the rise; the number of people diagnosed with depression increased by ~18% from 2005 to 2015^66^. Notably, rates of MDD correlate with modernization of society^67^; which may reflect the increased circadian disruption (i.e., light at night, shift work, and jet lag) or the interaction between circadian disruption and other environmental factors experienced in modernized countries.

There are few human studies that have examined the link between circadian disruption and MDD specifically, as most human data combine all types of depression. Nonetheless, studies examining the association between shift work and MDD have produced varied results^68–70^. For example, in a population of ~4000 South Koreans, the prevalence of MDD among shift/night workers was significantly increased relative to daytime workers^68^. Among a Brazilian cohort of ~36,000 workers, night shift work was significantly associated with MDD only in females^70^. No association was reported between shift work and MDD in a French study^69^. If all types of depression are combined, then a clear association emerges between shift work and depression^13,71–73^. A meta-analysis of 11 studies concluded that night shift workers are 40% more likely to develop depression relative to daytime workers^13^.

Human studies examining the effects of jet lag or social jet lag on MDD are sparse^74^. One such study, examined the amount of social jet lag (i.e., absolute difference between the midsleep on non-work days and midsleep on work days) in patients with MDD and healthy individuals. The authors concluded that there was no association between the amount of social jet lag and severity of depressive symptoms. Additionally, patients with MDD demonstrated no differences in social jet lag relative to healthy people^74^. However, in common with shift work, generalizing depression (i.e., combining all types of depression) results in a link between jet lag/social jet lag and depression^75–79^.

Human clinical data support a strong interaction between MDD and circadian processes. Symptoms of depression demonstrate diurnal variations; patients exhibit symptoms in a morning-worse or evening-worse pattern^80^. Patients with MDD typically express more severe symptoms in the morning, which is considered to be associated with a more severe form of depression^80^. Disruptions of biological rhythms underlie hallmarks of MDD; specifically, alterations in sleep/wake states (decreased latency to rapid eye movement sleep, concurrent with increased rapid eye movement sleep and reduced slow wave sleep), social rhythms, hormone rhythms (reduced amplitude in melatonin and cortisol rhythms), and body temperature rhythms (reduced amplitude and increase in nocturnal body temperature) are seen in patients with MDD^81^. Clinical studies demonstrate that the severity of MDD is correlated with the degree of misalignment of circadian rhythms^82^. Further, examination of circadian patterns of gene expression within postmortem brains of patients with MDD demonstrate reduced amplitude, shifted peaks, and altered phase relationship between genes, particularly in canonical clock genes^27^.

Most successful treatments of depression, bright light therapy, wake therapy, social rhythm therapy, and antidepressants (SSRIs, SNRIS, and agomelatine) directly affect circadian rhythms^83^. Morning bright light therapy phase advances the rhythm in melatonin and the degree of phase advancement is correlated with the amelioration of depressive symptoms^84^. Additionally, administration of antidepressants (SSRIs, SNIRs, and agomelatine [MT1, MT2 agonist and 5HT_2c_ antagonist) result in a phase advancement of circadian rhythms (i.e., melatonin, body temperature, activity, and cortisol rhythms)^85,86^. Wake therapy directly alters sleep-wake rhythms by increasing the amount of SWS, reducing the latency to REM sleep, and reducing the duration of REM sleep^87^. Thus, successful treatment of MDD with chronotherapies, as well as diurnal variation in symptoms provide evidence that circadian disruption may underlie this disease.

There is ample basic science evidence to support an association between disrupted circadian rhythms and depressive-like behavior. To assess the role of the SCN in regulating depressive-like behavior, Tataroglu and colleagues bilaterally lesioned the SCN and examined the effect on depressive-like behavior via forced swim tests^88^. Rats that received bilateral SCN lesions demonstrated reduced immobility. Thus, the authors concluded that SCN lesions have a protective effect in the induction of behavioral despair^88^. Similar conclusions were reached almost a decade earlier when Arushanyan and colleagues demonstrated reduced immobility during the forced swim test, in rats that received bilateral SCN lesions^89^. It may be difficult to draw definitive conclusions from these studies as testing of depressive-like behavior occurred during the inactive phase of the rats. SCN lesions likely disrupted the diurnal rhythm in activity; subsequent studies examining the role of the SCN in regulating depressive-like behavior have reached the opposite conclusion utilizing animals with an intact SCN^83,84^. Genetic disruption of circadian rhythms in the SCN via SCN-specific Bmal1-knockdown, or forced desynchrony of the SCN via exposure to a T22 light–dark cycle increased depressive-like behavior in mice and rats, respectively^90,91^.

Similar effects of increased depressive-like responses have been demonstrated in other rodent models of circadian disruption. Rats exposed to 8 weeks of constant light exhibit increased depressive-like behavior concurrent with loss of diurnal rhythms in activity, melatonin, and corticosterone^92^. Administration of agomelatine to rats exposed to 3 and 6 weeks of constant light prevented the increase in depressive-like behavior and restored diurnal corticosterone and melatonin rhythms respectively^93,94^. As with constant light, exposure to dim light at night induces depressive-like behavior in multiple species of rodents^58–60,95^. In female Siberian hamsters, exposure to 4 weeks of dim light at night (dLAN; 5 lux) increases depressive-like responses and neuroinflammation with concurrent decreases in dendritic spine density within the hippocampus^59^. Treatment with a dominant-negative TNF inhibitor prevented the increase in depressive-like behavior^59^. Additionally, diurnal rats exposed to dim light at night (5 lux) for 3 weeks demonstrated increased depressive-like behavior and reduced dendritic length within CA1 and dentate gyrus^58^. Acute (3 nights) exposure of mice to dLAN (5 lux) is sufficient to induce alterations in clock genes and increase depressive-like behavior^60^. However, studies have also reported no association between LAN and mood in C57Bl/6 mice suggesting a potential strain specific effect of LAN^96,97^. Taken together, there is substantial clinical and basic science evidence to support a link between circadian rhythm disruption and major depressive disorder.

### Circadian rhythm disruption and anxiety

Although several studies have suggested that night shift work and persistent jet lag provoke anxiety, more recent analyses suggest that the mood changes may reflect disturbed sleep, rather than disturbed circadian rhythms per se. For example, a longitudinal study of day shift workers without prior sleep disturbances who transitioned to rotating shift work schedules reported elevated anxiety along with disordered sleep^98^. Similarly, nurses with Shift Work Disorder display elevated anxiety scores on the Hospital Anxiety and Depression Scale^99^. However, rapid transitions to night shift work did not affect anxiety levels in a questionnaire study of nurses^100^. Jet lag, accomplished by undergoing a 7-h westward time shift by jet in five men preceding the study and, 1 month later, a 7-h eastward shift preceding was associated with disrupted sleep and elevated anxiety and depression scores, especially in simulated eastward travel^20^.

Studies of rodents revealed relationships among the circadian system and anxiety-like disorders. For instance, targeted disruption of canonical molecular clock components alerts anxiety-like behavior. Mice with a Δ19 mutation in the *Clock* gene display reduced anxiety-like behavior and are less fearful of aversive stimuli than wild-type mice^101^. Notably, clock regulates cholecystokinin (CCK) expression in the ventral tegmental area (VTA) and Δ19 mutation in the *Clock* gene is sufficient to induce manic-like behaviors^102^. In contrast, mice lacking both *Per1* and *Per2* display elevated anxiety-like behavior, whereas mice lack either *Per1* or *Per2* do not have altered anxiety-like responses^103^. Inhibition of *Per1/Per2* expression in the nucleus accumbens (NAc) of wild-type mice also produces anxiety-like behavior, suggesting a causal role for these core clock components in the NAc for regulating anxiety.

Other studies have investigated how environmental disruption of circadian rhythms (e.g., via exposure to light at night) contributes to the development of anxiety-like behavior. For example, housing adult rats chronically in constant light induces anxiety-like behavioral responses^92^. However, the effects of light as a circadian disruptor are inconsistent across species^104–108^, and may depend on the developmental window during which circadian disruption occurs, as well as the type of light (i.e. halogen, compact fluorescent, or light emitting diode) and its intensity^108,109^. For example, exposure to dim light at night during early development in mice increases adult anxiety-like responses^108,109^, whereas exposure of adult mice to light at night reduces anxiety-like responses^106^. Furthermore, glucocorticoid concentrations are often reduced in hamsters and unaltered in mice exposed to light at night compared to dark nights, suggesting that the affective behavioral responses to atypical lighting are not the result of elevated corticosterone^60,110,111^. Mice housed in 20-h light–dark cycles, a paradigm that disrupts circadian rhythms, display reduced dendritic length and complexity in neurons of the prelimbic prefrontal cortex, associated with anxiety^112^. Obviously, the extent to which light exposure alters sleep differs among species of diurnal and nocturnal rodents^56,113^. Together these data provide modest evidence in support of an association between circadian rhythm disruption and anxiety.

### Circadian rhythm disruption and bipolar disorder

Bipolar disorder (BD) is identified by cyclic extreme mood swings between mania and depression separated by periods of normal affect. This brain disorder is divided into four categories (in decreasing order of severity of the symptoms); Bipolar I, Bipolar II, Cyclothymic, and Other. These extreme mood episodes differ greatly from the typical behavior of the person, and are concurrent with significant changes in sleep, activity, and energy levels. BD is a genetic disorder, with 85–89% heritability^114^, however, no causal “smoking gun” gene has yet been identified. Genetic linkage studies have been equivocal^115^, yet modest associations have been reported between BD and multiple genes of the molecular circadian clock^116,117^. Additionally, treatment regimens for BD that normalize circadian rhythms have proven effective as treatments (see below), further implicating dysregulation in the circadian system in the pathology of this disease. Dysregulation of, or certain polymorphisms in, the afore associated genes involved in the circadian molecular clock may increase susceptibility to develop BD and also influence circadian phenotypes which could lead to relapse into episodes^117^. However, in a recent meta-analysis of 42 clinical studies on circadian rhythms and BD, the authors determined that, although circadian rhythm disruption is prevalent in BD, the cross-sectional research design of most studies precluded establishing a cause/effect relationship between circadian disruption and BD^118^. Thus, it remains undetermined whether circadian disruption is a primary pathophysiology of BD, or if it is secondary to other environmental and genetic factors. Nonetheless, a recent study has proposed the possibility of using circadian rhythms in buccal cell circadian clock gene expression and cortisol rhythms as biomarkers in BD patients for depression (phase delayed rhythms) and mania (phase-advanced rhythms)^119^.

Circadian disruption in the form of jet lag has been reported to induce bipolar episodes in susceptible people who fly across multiple time zones; east to west travelers with BD who then experience a phase delay in circadian rhythms at their destination are more likely to develop depression, whereas those traveling west to east who then experience a phase advance in their circadian rhythms are more likely to develop mania^75,120,121^. Disruption of circadian social rhythms, such as social jet lag, may also induce bipolar episodes. A major social disruptive event is associated with inducing mania, but not depression in BD patients^122^.

Historically, treatment for BD symptoms has been discovered by serendipity (lithium) or informed by treatments developed for other disorders with shared state, such as depression (described above). Early studies reported that BD patients have a ‘fast running’ circadian clock, likely leading to chronic circadian disruption; treatment with lithium, which slows the molecular circadian clock, ameliorates the symptoms and stabilizes circadian rhythmicity^123^. The approach of normalizing and stabilizing circadian rhythms, through lithium or other methods, has proven an effective therapy for ameliorating episodes of depression and mania, and for preventing relapse into these states^124,125^. Randomized placebo-controlled clinical trials have demonstrated that normalizing disrupted circadian rhythms with midday bright light therapy can resolve episodes of bipolar depression, whereas morning bright light therapy to treat depression in BP patients can induce mixed states^126,127^. Other clinical trials have reported that episodes of bipolar mania can also be treated effectively by normalizing disrupted circadian rhythms with enforced darkness; either through blue light blocking glasses^128^ or controlled environmental darkness^129^.

In sum, disrupted circadian rhythms appear to be both a state marker and a trait of BD. These disruptions of circadian rhythms can arise either via internal desynchrony or environmental desynchrony and can both predispose one to BD, as well as induce bipolar episodes, dependent on the phase relationship between the internal and external circadian rhythms. Resynchronization and normalization of circadian rhythms (chronotherapy) has proven effective in prevention and treatment of bipolar episodes.

There is a paucity of animal studies of BD mainly because there are no animal models that satisfy the validity criteria for modeling this complex multifactorial disorder^130–133^. Most animal models of BD are endophenotype-based and probe the various states of BD from depression (see above) to mania-like behaviors (for a detailed review see Logan and McClung^133^, yet none have adequately captured the hallmark of BD: state switching. A recent study proposed a mouse model of state switching based on photoperiod wherein it was reported that mice with reduced dopamine transporter (DAT) expression differentially express increased depressive-like or mania-like behaviors dependent upon day length^134^. However, the validation of DAT deficient mice as a model of state switching in BD remains controversial^135^. Regardless, altered dopaminergic signaling has been implicated in bipolar mania. One of the most well-established models of bipolar mania, the *Clock*Δ19 mutant mouse, has a fundamentally disrupted molecular circadian clock and restoration of a functional molecular circadian clock to the VTA, likely restoring dopaminergic regulation, ameliorates the mania-like behaviors^101^. Given that *Clock*Δ19 mutant mice have a genetically disrupted circadian clock and already recapitulate many mania-like behaviors based on their inherent internal circadian desynchrony, this model does not lend itself well to studying the effects of environmental circadian disruption on bipolar mania.

Circadian disruption by sleep deprivation can induce a manic-like phenotype in mice (increased aggression and hyperlocomotion), yet these effects are transient and behaviors return to baseline within 24 h^136^. Studies on circadian disruption in ICR mice, by inverting the LD cycle for 3 days, have reported that approximately half of the mice fail to recover normal sleep-wake cycles within 6 days after re-exposure to normal lighting conditions. The mice that took longer to re-entrain normal sleep-wake rhythms after circadian disruption also displayed greater quinpirole-induced hyperactivity, indicative of a more mania-like state, however depressive-like behavior (forced swim test) was not affected^137,138^. Based on these studies and human clinical data, the pathophysiology of environmental circadian disruption underlying bipolar mania may be via altered protein kinase c activity affecting neuronal signaling in frontal and limbic brain regions^139^.

### Circadian rhythm disruption and schizophrenia

Schizophrenia (SZ) is a fairly rare (~0.3–0.7 % lifetime prevalence), but severely disabling, mental disorder that typically emerges in the second or third decade of life^140,141^. The high heritability of SZ (~80%) suggests a substantial genetic risk, however, the lower concordance between monozygotic twins (~50%) demonstrates that other factors, such as environment, epigenetic modifications, or nutrition, impact illness risk^142^. SZ is characterized by positive symptoms (delusions, hallucinations, thought disorders, and movement disorders), negative symptoms (flat affect, anhedonia, alogia, reduced sociality, and avolition), and cognitive symptoms (impaired executive function, attentional deficits, and impaired working memory). Furthermore, the severity of SZ symptoms has been associated with the extent of sleep or circadian rhythm disruption^143–145^. Indeed, circadian disruption is a common prodrome of SZ^145^, although the nature of the relationship remains unclear, in part because circadian disruption has been studied less extensively in SZ than other mood disorders. There are case studies reporting the emergence and relapse of SZ/schizoaffective psychosis among individuals who have traveled across time zones, and therefore may be experiencing jet lag^146,147^, but no large scale, controlled studies have examined whether jet lag, social jet lag, simulated jet lag, or other forms of circadian disruption precipitate or exacerbate SZ symptoms.

In contrast, studies have demonstrated altered diurnal rhythms in subjects with SZ. Using postmortem brain tissues, Seney and colleagues^148^ demonstrated that subjects with SZ lost rhythmicity in most genes within the prefrontal cortex that were rhythmic in healthy controls. However, subjects with SZ gained rhythmicity in completely different set of genes not seen in healthy controls (e.g. mitochondrial related genes). Additionally, genes that have previously been implicated in SZ (i.e. BDNF and GABAergic-related transcripts) were differentially expressed in SZ subjects that died during the night^148^. Further, several studies have documented alterations among SZ patients in two key endocrine transducers of the circadian clock: melatonin and cortisol. As described above, melatonin typically begins to rise with the onset of darkness and peaks several hours later, whereas cortisol concentrations are typically low over night and begin to rise prior to waking. Studies of individuals with SZ who were not currently taking medication have revealed alterations in several aspects of the melatonin rhythm, including reduced nighttime melatonin concentrations^149–151^, blunted rhythmic amplitude^149,150^, and phase advance of the typical melatonin acrophase^152^. Among medicated SZ patients, a subset of individuals exhibit a phase delay^153^ or phase advance^154^ of an otherwise typical melatonin rhythm, whereas others experience a blunting of the rhythm with or without a phase shift^143^. Regardless of the direction, a “phase-shift” in the melatonin rhythm can result in uncoupling from the environmental day/night cycle, in turn desynchronizing physiological processes and the behaviors they subserve. Indeed, the breakdown of the typical relationship between the melatonin rhythm and the sleep-wake cycle, has led some researchers to propose that the sleep promoting properties of endogenous melatonin are compromised in SZ^155^. However, others have proposed that the social withdrawal that often occurs in SZ may contribute to circadian disruption by limiting exposure to zeitgebers that are important for keeping the clock entrained to the 24 h day^143^.

In contrast to melatonin, the cortisol circadian rhythm appears to remain more or less intact in SZ^150–152^, although concentrations are often elevated relative to healthy controls^156,157^. Interestingly, hyper-reactivity of the HPA axis is observed in both individuals with SZ and individuals at high genetic risk for SZ, which may support the model of stress vulnerability in SZ^158^. Furthermore, altered regulation of the HPA axis in SZ is predictive of more severe psychotic and motor symptoms^159,160^. Fortunately SZ-associated HPA axis hyperactivity is responsive to treatment; 4 weeks of treatment with olanzapine produces cortisol concentrations that are comparable to healthy controls^161^.

As mentioned, there is a strong genetic component to SZ, and given the extensive disruption of circadian rhythms in this mood disorder, it is not surprising that several core clock genes have been implicated. In a study that examined 276 single nucleotide polymorphisms (SNPs) at 21 circadian genes, significant associations were detected for 8 SNPs from 4 circadian-related genes (*Retinoic acid-related orphan receptor* (*RORβ*), *Per2, Per3*, and *Neuronal Pas domain protein 2*), however, no associations remained significant after correcting for multiple comparisons, suggesting these individual polymorphisms in clock genes are unlikely to confer sizeable (>1.5 OR) risk for SZ^162^. An earlier and smaller study also identified modest associations for PERIOD3 and TIMELESS in patients with SZ^163^. Data collected from cultured skin and blood cells also support the conclusion of disrupted circadian rhythms in SZ. Specifically, fibroblasts enriched from skin biopsies of SZ patients exhibit loss of rhythmic expression of CRY1 and PER2 compared to cells from healthy controls, while rhythms in BMAL1, REV-ERBα persist in a manner comparable to the cells from healthy individuals^164^. Similarly, mononuclear blood cells from SZ patients experiencing their first episode of psychosis, have decreased expression of CLOCK, PER2, and CRY1 relative to non-SZ individuals^164^.

Another gene that has been implicated in both SZ and circadian organization is immediate *early gene growth response 3* (*Egr3*)^162,165–168^. Additional support for this candidate gene is provided by Egr3-deficient mice (−/−), which display behaviors that are reminiscent of SZ and sleep less than WT controls, even though the circadian organization of the sleep-wake cycle remained intact^169^.

In humans, duplication of the vasoactive intestinal polypeptide receptor *(Vipr2)* is associated with increased risk for SZ^170,171^. Mice that are deficient in *Vipr2* exhibit cognitive deficits associate with hippocampal dependent associative memory- which is reminiscent of SZ^172^. Under a 12:12 LD cycle, the VPAC2-KO mice adrenal clock gene rhythm and corticosterone rhythm was phase-advanced compared to WT mice and when the lighting was changed to constant darkness, the adrenal clock genes and corticosterone secretion became arrhythmic^173^. These data support the role of VIP in synchronization of internal rhythms. Importantly, the low concordance of SZ in monozygotic twins suggest that alterations in the core clock genes are not likely to be the cause of SZ, but they may affect disease progression, symptom severity, or efficacy of treatment.

### Future directions

Although light at night from modern electronic devices may be responsible for the circadian disruption affecting the psychiatric diseases discussed above, these devices might also provide the data to help resolve some disease states and improve outcomes. A recent study analyzing digital logon data from nearly 15,000 students reported that circadian disruption (social jet lag) negatively affects learning outcomes, and furthermore that these data could be used to time educational activities to minimize impairments in performance caused by circadian disruption at the individual and population levels^174^. With the prevalence of hand-held and wrist-worn mobile devices today, it may also be possible to leverage data from these devices to predict and monitor psychiatric state of users. Indeed, passively collected data from mobile phones can be used to monitor and predict real time behavioral indicators of depression and PTSD^175^. Furthermore, passively collected keystroke metadata may also be used to predict state changes found in BD^176^. In future studies, it might be informative to consider leveraging data from the devices’ sensors to monitor ambient light, at eye level, in association with the other data collected to investigate whether exposure to light at night is a predictive cofactor in affective disorder state.

Additionally, future studies should focus on expanding both the clinical and basic science literature in relation to circadian disruption and behavioral health. There are numerous studies in humans examining the effects of shift work on behavioral and psychiatric health, however, the number of studies examining the effects of jet lag on mental health are modest. In 2017, commercial airlines carried more than 4 billion passengers (>50% of the world’s population). Hence, modern air travel has greatly increased the likelihood of experiencing disruption in circadian rhythms. Clinical and basic science research should reflect the increased prospect of experiencing circadian disruption via jet lag. Studies examining the effects of social jet lag on mental health are even more scarce. As noted, ~80% of the population suffers from social jet lag. Thus, it is essential to understand the consequences of social jet lag on behavioral health and appropriately manage them. Ultimately, the expansion of research in this field will broaden our knowledge and may result in novel treatments to improve patients’ quality of life and outcomes.

## Conclusion

Altered circadian rhythms are commonly reported among individuals with several psychiatric disorders, including major depressive disorder (MDD), bipolar disorder (BD), anxiety, and schizophrenia (SZ). However, the nature of the relationship between circadian rhythm disruption and psychopathology is poorly understood. The vast majority of clinical data are correlational. Thus, it remains unknown whether the relationship reflects: (1) causation in which circadian disruptions predisposes individuals to developing mood disorders, (2) causation in which the manifestation of mood disorder leads to circadian disruption, or (3) an absence of causation in which the association between circadian disruption and mood disorders reflects commonalities in underlying physiological processes^177^. However, rodent studies have demonstrated that even among healthy animals, experimentally induced disruptions of circadian rhythms can lead to affective changes. Targeted resynchronization of circadian rhythms improves symptoms of mood disorders. In sum, while circadian disruption may not be the sole cause of mood disorders, it may elicit or exacerbate symptoms in individuals with a predisposition for mental health disorders.
